# Changes in Carboxy Methylation and Tyrosine Phosphorylation of Protein Phosphatase PP2A Are Associated with Epididymal Sperm Maturation and Motility

**DOI:** 10.1371/journal.pone.0141961

**Published:** 2015-11-16

**Authors:** Tejasvi Dudiki, Suraj Kadunganattil, John K. Ferrara, Douglas W. Kline, Srinivasan Vijayaraghavan

**Affiliations:** Department of Biological Sciences, Kent State University, Kent, Ohio, United States of America; Faculty of Medicine, BELGIUM

## Abstract

Mammalian sperm contain the serine/threonine phosphatases PP1γ2 and PP2A. The role of sperm PP1γ2 is relatively well studied. Here we confirm the presence of PP2A in sperm and show that it undergoes marked changes in methylation (leucine 309), tyrosine phosphorylation (tyrosine 307) and catalytic activity during epididymal sperm maturation. Spermatozoa isolated from proximal caput, distal caput and caudal regions of the epididymis contain equal immuno-reactive amounts of PP2A. Using demethyl sensitive antibodies we show that PP2A is methylated at its carboxy terminus in sperm from the distal caput and caudal regions but not in sperm from the proximal caput region of the epididymis. The methylation status of PP2A was confirmed by isolation of PP2A with microcystin agarose followed by alkali treatment, which causes hydrolysis of protein carboxy methyl esters. Tyrosine phosphorylation of sperm PP2A varied inversely with methylation. That is, PP2A was tyrosine phosphorylated when it was demethylated but not when methylated. PP2A demethylation and its reciprocal tyrosine phosphorylation were also affected by treatment of sperm with L-homocysteine and adenosine, which are known to elevate intracellular S-adenosylhomocysteine, a feedback inhibitor of methyltransferases. Catalytic activity of PP2A declined during epididymal sperm maturation. Inhibition of PP2A by okadaic acid or by incubation of caudal epididymal spermatozoa with L-homocysteine and adenosine resulted in increase of sperm motility parameters including percent motility, velocity, and lateral head amplitude. Demethylation or pharmacological inhibition of PP2A also leads to an increase in phosphorylation of glycogen synthase kinase-3 (GSK3). Our results show for the first time that changes in PP2A activity due to methylation and tyrosine phosphorylation occur in sperm and that these changes may play an important role in the regulation of sperm function.

## Introduction

The serine-threonine protein phosphatase, protein phosphatase 2A (PP2A), is constitutively expressed in all eukaryotes. This phosphatase is implicated in diverse functions such as metabolism, DNA replication, transcription, RNA splicing, translation, cell-cycle progression, morphogenesis, development, and transformation [1,2]. Protein phosphatase 2A is composed of three subunits, the catalytic subunit PP2A-C, scaffolding subunit PP2A-A, and the regulatory subunit PP2A-B. The catalytic subunit has two isoforms, PP2A-Cα and PP2A-Cβ, which are 97% identical with a conserved C-terminus end. The A (or PR65) subunit is the scaffolding or the structural subunit that anchors the other two subunits together. It also exists in two isoforms, PP2A-Aα and PP2A-Aβ, both with a molecular weight of 65kDa. PP2A is known to exist as a hetero-dimer or a hetero-trimer. The 36 kDa catalytic subunit (PP2A-C) is tightly bound to the 65 kDa A scaffolding subunit (PR65) forming a heterodimer (AC). This dimer binds to a regulatory (B) subunit from one of four gene families designated as B/PR55, B'/PR61/B56, B'', and B‴ to form a hetero-trimer (ABC). The B and B' family contains α, β, γ and δ isoforms with the B' family including an extra ε isoform. The B'' family includes the isoforms PR48, PR59 and the splice variants PR72 and PR130, while the B‴ family includes striatin and SG2NA [2]. Both A and B subunits have been shown to play a role in controlling the substrate specificity of PP2A. The B subunits appear to determine substrate specificity and sub-cellular localization of PP2A [3–6].

The catalytic subunit of PP2A (PP2A-C) can be covalently modified at the C-terminus by carboxymethylation [7] and tyrosine phosphorylation [8]. Leu 309, the C-terminal amino acid residue of PP2A, is highly conserved among all mammals [9]. Carboxymethylation of the Leu 309 is thought to be essential for B subunit binding and formation of the hetero-trimeric holoenzyme complex [10–12]. Methylation is catalyzed by a protein phosphatase methyl-transferase (LCMT1 or PPMT1) and demethylation by a protein phosphatase methyl-esterase (PME1) [7,13] which are each specific for PP2A as a substrate. Methylation of the PP2A catalytic subunit was observed to increase its activity toward phosphorylase *a*, a substrate which was used to measure its catalytic activity [9]. However, recent evidence suggests that methylation of the catalytic subunit affects PP2A activity by enhancing the association of the regulatory B subunits to the core AC dimer [11]. Phosphorylation of the catalytic subunit of PP2A (PP2A-C) on Tyr307 reduces its catalytic activity [8,14]. Therefore enzymatic activity following methylation of the catalytic subunit is regulated depending on the nature of the B-subunit in the holoenzyme and phosphorylation of the catalytic subunit. Among the protein kinases known to phosphorylate PP2A-C on tyrosine include p60v-src from *v-scr* transfected fibroblasts, p56lck from transformed T cells, and the receptor kinases, for epidermal growth factor and insulin [8]. Reversible PP2A methylation is essential for life since targeted disruption of PME or LCMT1 in mice is embryonic lethal [15,16].

Testicular spermatozoa in mammals are immotile and lack the ability to fertilize eggs *in vitro*. Sperm motility initiation and fertilizing ability develop during their passage through the epididymis [17]. Despite a large body of knowledge, we do not completely understand how changes in the epididymis lead to metabolic competence, motility development, and the ability of spermatozoa to bind and fertilize the egg. The capacity for motility already exists in testicular sperm, demonstrated by the fact that motility can be induced in demembranated testicular and caput epididymal sperm in the presence of ATP, cAMP, appropriate calcium levels, and proper pH [18,19]. It is well known that motility stimulation can be affected by cAMP-mediated PKA activation [20–22]. A role for PKA necessarily implies a function for a protein phosphatase. Protein phosphatases can significantly modify and restrict PKA action. Inhibition of protein phosphatase activity results in initiation and stimulation of motility [23,24]. Inclusion of protein phosphatases in reactivation media prevents PKA-mediated motility initiation in demembranated sperm [24,25].

We have shown that the testis isoform of protein phosphatase 1, PP1γ2, is essential for spermatogenesis and is also involved in sperm motility regulation [23,24]. Immotile caput epididymal sperm have high PP1γ2 activity whereas motile caudal epididymal sperm have low PP1γ2 activity. Furthermore, inhibition of phosphatase activity with okadaic acid and calyculin A results in initiation of motility in immotile caput spermatozoa [23]. Okadaic acid and calyculin A also stimulate motility and induce changes in flagellar beat parameters in already motile caudal sperm. While we had previously reported the identification of the 36-kDa catalytic subunit of PP2A in bovine spermatozoa [23], the role of PP2A in sperm function was not explored. Here we report for the first time that changes in PP2A catalytic activity, methylation, and tyrosine phosphorylation occur during the passage of spermatozoa through the epididymis. Demethylation of PP2A results in a change in sperm motility characteristics. Inhibition of PP2A is unable to initiate motility in immotile caput epididymal sperm but is able to stimulate motility of motile caudal sperm. Our studies show that PP2A, regulated by methylation, may play an important role in epididymal sperm maturation and in the regulation of motility characteristics of mature sperm.

## Materials and Methods

### Sperm extract preparation

Mature bull testis were obtained from Duma Meats, a local commercial slaughter house within couple of hours of sacrifice. Spermatozoa were isolated from proximal caput, distal caput and caudal epididymis and washed twice in Hepes buffer, pH 7.0 (10 mM Hepes, 135 mM NaCl, 5 mM KCl, 5 mM MgSO_4_) [24]. Sperm pellets were adjusted to a volume of 10^8^ sperm/ml by resuspension in homogenization buffer with protease inhibitors (HB+; 10 mM Tris [pH 7.2] containing 1 mM EDTA, 1 mM EGTA, 10 mM benzamidine-HCl, 1 mM PMSF, 0.1 mM *N-p*-tosyl-L-phenylalanine chloromethyl ketone [TPCK], 0.1% (V/V) β-mercaptoethanol and 1 mM sodium orthovanadate). The sperm suspension was sonicated on ice with three 10-sec bursts of a Microson ultrasonic cell disruptor (Misonix Inc.) set at level three. The suspension was then centrifuged at 16,000 x g for 20 min. at 4°C. The supernatants were supplemented with 5% glycerol and stored at -20°C.

### Pull-down of PP2A with Microcystin-Agarose and demethylation of PP2A

Microcystin-agarose beads (Upstate) were washed two times with an equal volume of HB+ buffer supplemented with 5% BSA to prevent nonspecific binding. The Microcystin-agarose beads were mixed with 100 μl sperm extract at 4°C with rotation overnight. Following this incubation the flow-through (unbound) fraction was collected by centrifugation. The bead pellet (bound PP2A) was washed by centrifugation twice with TTBS (0.2 M Tris [pH 7.4], 1.5 M NaCl, 0.1% thimerosol and 0.5% Tween 20) and two times with HB+ buffer. The flow-through and pellet fractions were boiled with Laemmli sample buffer and stored at -20°C for Western blot analysis.

For alkali mediated PP2A demethylation, PP2A in sperm extracts was first bound to microcystin. A 10 μl volume of microcystin-agarose bead slurry (prepared as described above) was incubated with sperm extracts prepared from the proximal caput, distal caput and caudal regions of the epididymis. The extracts were incubated for 150 min, washed in TTBS wash buffer (0.2 M Tris [pH 7.4], 1.5 M NaCl, 0.1% thimerosol and 0.5% Tween 20) twice and in HB+ once. The washed microcystin-agarose beads were then resuspended in 30μl of HB+. Microcystin bound PP2A from each of these three regions was divided into two equal aliquots; one part was treated with 0.2 M NaOH while the other was left untreated as control. After 8 minutes at 4°C the alkali was neutralized with 0.2 M Tris-HCl (pH 6.8). An equal volume of neutralized alkali was added to the control suspension [26]. Microcystin beads were pelleted and boiled with Laemmli sample buffer followed by Western blot analysis.

### Western blot analysis

Sperm extracts, denatured by boiling for 3 min in Laemmli sample buffer, were separated by SDS-gel electrophoresis using 12% polyacrylamide slab gels in a Mini-Protean II system (Bio-Rad Laboratories). After electrophoresis, proteins were electrophoretically transferred to Immobilon-P, PVDF membrane (Millipore Corp). Nonspecific protein binding sites on the membrane were blocked with 5% nonfat dry milk in TTBS (0.2 M Tris [pH 7.4], 1.5 M NaCl, 0.1% thimerosol and 0.5% Tween 20). The blots were washed with TTBS and then incubated overnight with primary antibody at 4°C. The following primary antibodies were used during this study: PP2A-C demethyl-sensitive antibody 4b7 (mouse monoclonal antibody raised against residues 295–309 of PP2A, Upstate) (1:2000), anti-PP2A-C (mouse monoclonal against residues 153–309, BD Biosciences) (1:2000), anti-PP2A-C (raised in rabbit against residues 288–306, commercially prepared) (1:1000), anti-phospho-PP2A-C Y307 (rabbit monoclonal, Epitomics) (1:500), anti-PP1γ2 (commercially prepared) (1:5000) [23], anti-PME1 (rabbit polyclonal, Millipore) (1:1000), Anti LCMT1 (mouse monoclonal, Millipore) (1:1000), anti-phospho GSK3 α/β (rabbit polyclonal against Ser 21/9, cell signaling) (1:2000) and anti-β-tubulin (mouse monoclonal, Zymed) (1:2000). After washing with TTBS, the blots were incubated with the appropriate secondary antibody (Amersham) conjugated to horseradish peroxidase at 1:5000 dilution for 1 hr at room temperature. Blots were then washed with TTBS twice 15 min each and four times 5 min each and then developed with enhanced chemiluminescence (Amersham).

### Protein phosphatase assay

Radio-labeled phosphorylase *a* was used as a substrate to measure catalytic activity of PP2A [23]. The substrate and sperm extracts were incubated (in a total volume of 30 μl) at 30°C with or without inhibitors for 15 min. Recombinant protein phosphatase Inhibitor 2 (I-2) (New England BioLabs) was used to inhibit PP1γ2 and 2nM Okadaic acid to inhibit PP2A. The reaction was terminated by addition of 90 μl 20% trichloroacetic acid (TCA) and incubation on ice. Samples were then centrifuged for 5 min. at 12,000 x g. Supernatants were analyzed for ^32^P released from phosphorylase *a*. Enzyme activity is expressed in mmol of PO4 released/minute/2x10^5^ sperm.

### Motility analysis of sperm

Sperm from the caudal epididymis were isolated and washed twice by centrifugation with HEPES buffer. Sperm were then incubated at 37°C in HEPES containing 20mM glucose and 20% BSA for control measurements and along with1 mM L-homocysteine in addition to 1 mM adenosine to elevate intracellular S-adenosylhomocysteine (SAH). For motility determination, samples were taken from sperm preparations at a concentration of 2X10^7^ sperm/ml. Motility was analyzed on 20 μm capillary microscope slides by the computer assisted analyzer (CASA) of Hamilton Thorne Biosciences installed with the CEROS version 12.2g software. Three random fields were chosen for each sample and motility was recorded for 90 frames at 60 frames per second. The data was analyzed with the following settings: minimum contrast 30, minimum cell size 4 pixels, static cell size 8 pixels, static cell intensity 60, low size gate 0.17, high size gate 2.26, low intensity gate 0.35, high intensity gate 1.84, minimum static elongation gate 0, maximum static elongation gate 90, minimum VAP 50μ/s, minimum STR 50%, VAP cut off 10μ/s and VSL cut off 10μ/s.

## Results

### Changes in PP2A methylation and tyrosine phosphorylation in bovine epididymal spermatozoa

**Fig 1:**
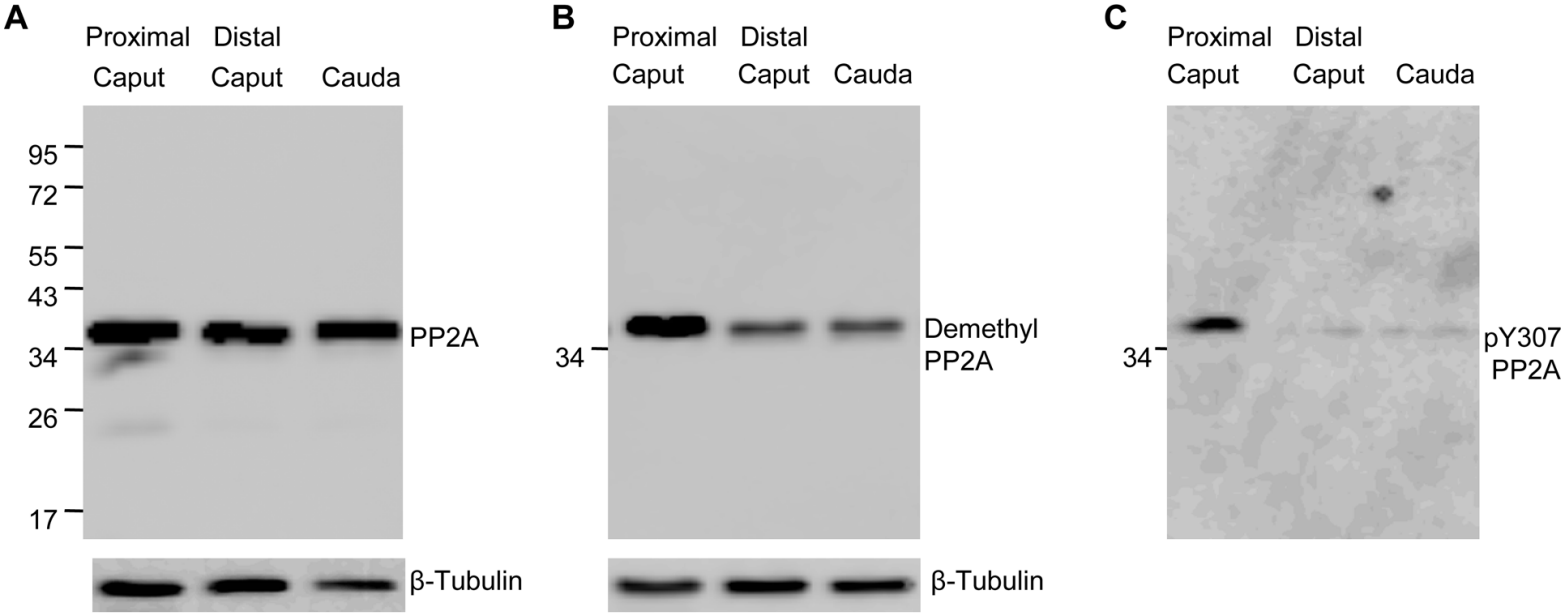
Western blot analysis of PP2A in sperm. **(A)** Presence of immunoreactive PP2A in sperm (2X10^6^sperm/lane) from different regions of epididymis. The blot was probed with PP2A antibodies that recognize PP2A irrespective of its methylation or tyrosine phosphorylation. The same blot was re-probed with anti β-Tubulin antibody as loading control. (**B)** Western blot with the same extracts used in panel A showing the methylation status of PP2A in sperm extracts (2X10^6^sperm/lane) from different regions of the epididymis, using anti-demethyl PP2A antibody. The blot was re-probed with anti β-Tubulin antibody as loading control for both B and C panels. (**C)** A duplicate blot of panel A and B (same extracts) but with higher sperm number (4X10^6^) loaded in each lane showing tyrosine phosphorylation determined by reactivity with anti-PP2A Y307 antibody.

Western blot analysis of soluble extracts of sperm isolated from proximal caput, distal caput, and caudal regions of the epididymis when probed with anti-PP2A antibody each showed one immunoreactive band at ~36 kDa, presumably due to PP2A. There was equal PP2A immunoreactivity in the three extracts obtained from each region (Fig 1A). Little or no PP2A immunoreactivity was observed in pellets of sperm sonicates. We next determined the methylation status of the PP2A catalytic subunit in sperm from the three regions of the epididymis. A mouse monoclonal antibody anti-PP2A 4B7 raised against the amino acid sequence (RGEPHVTRRTPDYFL) corresponding to residues 295–309 in human PP2A-C is demethyl-sensitive. This antibody is documented to react with PP2A-C in its demethylated form, that is, when the terminal leucine residue is not methylated [27]. This antibody is referred as “demethyl-sensitive antibody or anti-demethyl PP2A” in this report. The demethyl-sensitive 4B7 antibody detects a band at ~36 kDa in bovine sperm extracts (Fig 1B) from all three regions of the epididymis. However, based on densitometry analysis from four sets of experiments, immunoreactivity to the demethyl-sensitive antibody was on an average threefold higher intensity in proximal caput sperm compared to that in extracts of sperm from distal caput and caudal regions of the epididymis. This indicates that PP2A is less methylated (more demethylated) in sperm from the proximal caput epididymis and that it is more methylated in sperm from the distal caput and caudal epididymis. Each of the experimental blots included immune detection of tubulin as a protein loading control.

Since PP2A, in somatic cells is known to undergo tyrosine phosphorylation [11], we next examined whether a similar situation existed with sperm PP2A. Western blot analysis was performed using a rabbit monoclonal antibody specific to tyrosine phosphorylated (Tyr307) PP2A. Immunoreactivity to this antibody revealed that PP2A in proximal caput but not distal caput and caudal sperm extracts was tyrosine phosphorylated (Fig 1C).

### Microcystin pull-down and alkali treatment of sperm PP2A

**Fig 2:**
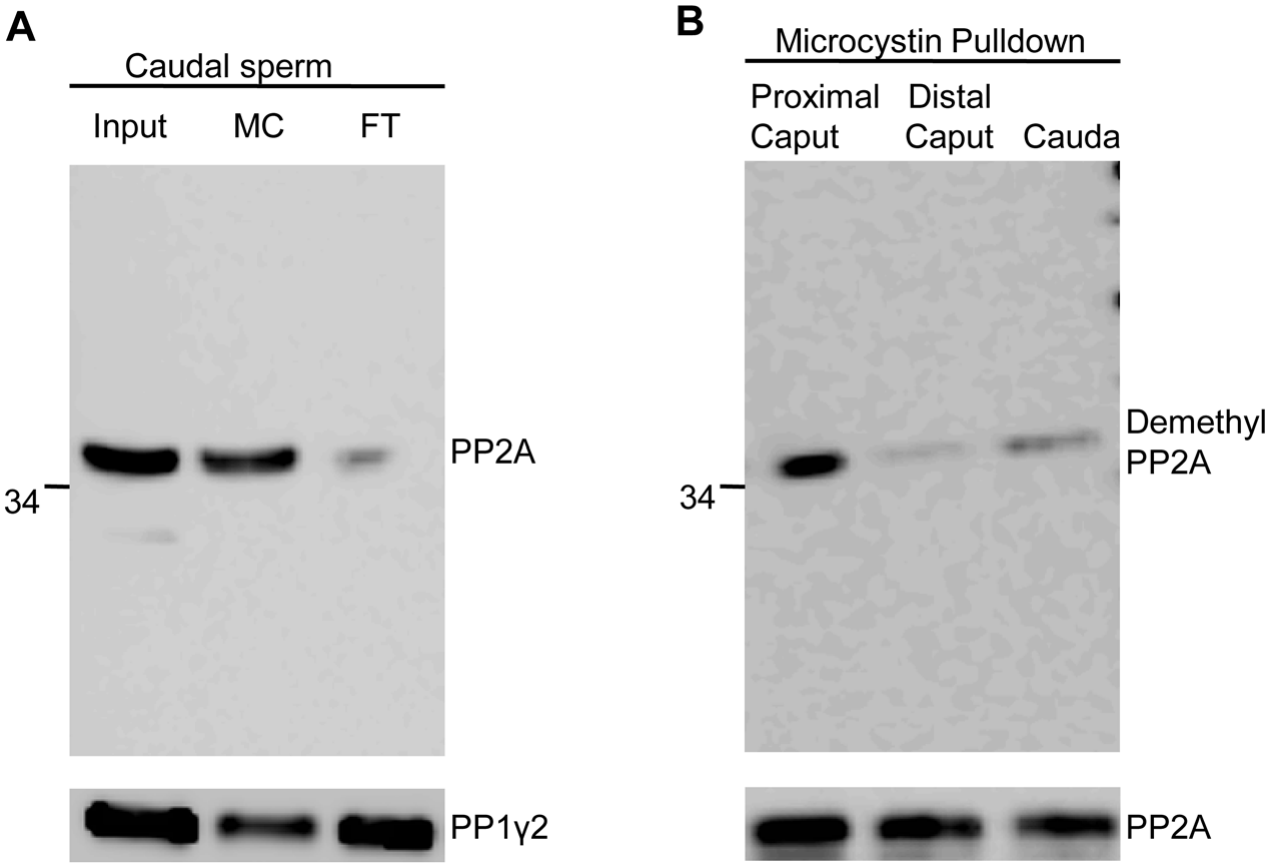
Microcystin pulldown of sperm PP2A. **(A**) Caudal sperm extracts (2X10^8^ sperm/ml) were incubated with microcystin- sepharose beads. The microcystin- bound proteins were eluted by boiling with Laemmli buffer and analyzed by Western blot with Anti-PP2A antibody (MC). 10μl of the input caudal sperm extract was loaded as a positive control (Input). Flow through (FT) obtained following the pull down, *i*.*e*. sperm extract left behind after incubation with microcystin sepharose beads, shows negligible levels of PP2A at the same control input loading volume. The same extracts were run as a duplicate blot and probed with Anti-PP1γ2 as a control for microcystin pull down. (**B)** Extracts from 5X10^7^ sperm from each region of epididymis were subjected to microcystin pull down. Equal amounts of the microcystin bound proteins boiled in SDS- sample buffer were loaded in each lane and probed with anti-demethyl PP2A antibody. A duplicate blot probed with anti-PP2A antibody as control.

Data in Fig 1 show that PP2A is present in equal amounts in developing epididymal sperm but it appears to be more methylated in sperm isolated from distal caput and caudal regions compared to sperm from proximal caput region of the epididymis. Microcystin pull down was used to further confirm that the immunoreactive bands seen in Western blot of sperm extracts are indeed due to PP2A. The cyclic peptide microcystin, a potent phosphatase inhibitor, covalently binds to the catalytic subunits of PP1 and PP2A [28,29]. Microcystin was able to almost completely pull-down PP2A, but not PP1γ2, from sperm extracts (Fig 2A). The inability of microcystin to completely pull down PP1γ2 in sperm extracts is previously documented [30]. Differences in immunoreactivity to the demethyl-sensitive PP2A-C antibody seen in sperm extracts are also observed in Western blots of PP2A isolated by microcystin, indicating again that PP2A is less methylated in proximal caput sperm (Fig 2B).

**Fig 3:**
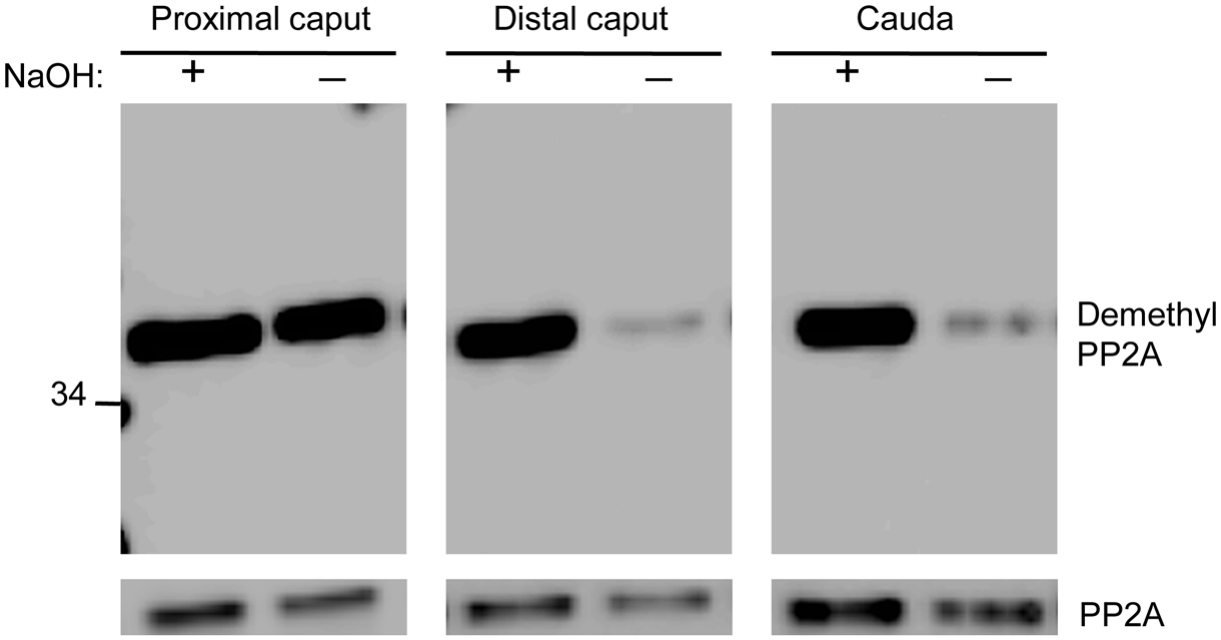
Demethylation of PP2A induced by alkali treatment. Microcystin beads incubated with sperm extracts from proximal caput, distal caput and caudal regions of epididymis were subjected to NaOH treatment (+). Equal amounts incubated without NaOH are untreated controls (-). Details of the procedure are described in Materials and Methods. Different volumes were loaded in Western blot for each epididymal region, hence shown as separate panels. Duplicate blots were processed, one was probed with anti-demethyl PP2A antibody and the other was probed with anti-PP2A antibody.

Though demethyl PP2A antibodies are well characterized, we sought to determine by an independent method that sperm PP2A methylation is different in the three regions of the epididymis. Carboxy-methyl groups in proteins can be removed by sodium hydroxide treatment [16,31]. PP2A was first isolated from extracts of sperm from proximal caput, distal caput and caudal regions of the epididymis with microcystin-agarose. Microcystin-bound PP2A was then treated with 0.2 N NaOH. The extracts treated with NaOH were then neutralized with HCl. The methylation status of PP2A in control and NaOH treated samples was determined by Western blot analysis. Result presented in Fig 3 shows that sodium hydroxide treatment increased immuno-reactivity of PP2A to the demethyl-PP2A antibody in sperm extracts from distal caput and caudal regions, presumably due to demethylation of PP2A. Extracts from proximal caput epididymis were unaffected by sodium hydroxide because PP2A in this extract was not methylated. Immuno-reactivity to the methyl-insensitive antibody was the same in alkali-treated and control samples showing equal amounts of PP2A in the samples.

### Detection of LCMT1 and PME1 in sperm

**Fig 4:**
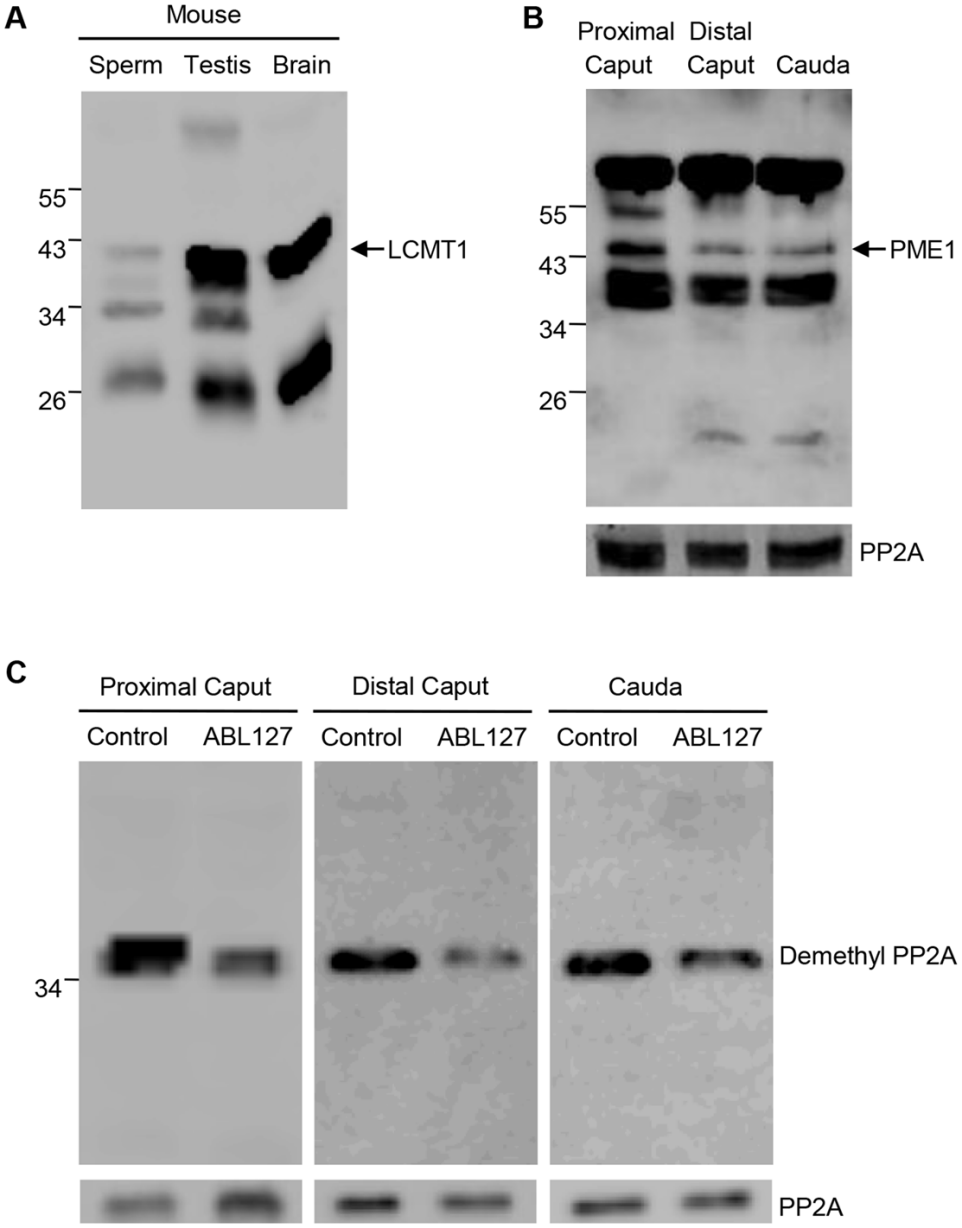
LCMT1 and PME1 in sperm. **A)** Western blot showing detectable levels of LCMT1 in mouse whole sperm extracts prepared in 1%SDS and testis extracts. Mouse brain extract was loaded as positive control for Anti-LCMT1 antibody. **B)** Presence of immunoreactive PME1 at ~43kd in HB+ sonicated soluble bull sperm extracts from all three regions of epididymis is shown with Anti-PME1 antibody. The blot was stripped and probed with Anti-PP2A antibody to show equal protein loading. **(C)** Sperm isolated from the three regions of the epididymis were incubated with DMSO (control) or 500 nM ABL127 for 1hr. Following this incubation, sperm extracts were prepared and analyzed by Western blot with anti-demethyl PP2A antibody. The first panel shows 10^6^ proximal caput sperm/lane at 10 seconds exposure. The remaining panels show 2X10^6^ distal caput or caudal sperm/lane at 60 seconds exposure. A duplicate blot probed with Anti-PP2A is used as loading control.

The enzyme PP2A methyl transferase, LCMT1 responsible for transferring the methyl group from S-adenosyl methionine specifically to PP2A is detected by Western blot analysis of mouse testis and sperm extracts probed with Anti-LCMT1 antibody at ~38Kd (Fig 4A). However, no immunoreactive band could be detected in bull testis or sperm soluble extracts. This could be explained by the inability of the antibody to recognize bull LCMT1, as the antibody was raised against 6His-tagged full-length mouse PP2A-methyltransferase/LCMT1 antigen. The bull LCMT1 differs by 8 amino acids compared to human or mouse. Mouse brain extracts known to contain high levels of LCMT1 were used as positive controls [32]. PP2A methyl esterase PME1 responsible for removal of methyl group from methylated PP2A with a molecular weight of approximately 43kd was detected in Western blots of bovine sperm from all three regions of the epididymis (Fig 4B). Corresponding with high levels of demethylated form of PP2A, we also observed higher levels of PME1 in sperm from proximal caput compared to sperm from other regions of epididymis. The Anti-LCMT1 and Anti-PME1 antibodies in addition to detecting LCMT1 and PME1 also cross reacted with other proteins. ABL127 a PME1 inhibitor was shown to increase methylation of PP2A in HEK293 cells [33]. To confirm the presence of PME1 in bull sperm we incubated sperm with ABL127 (500nM) or DMSO (control) for 1hour to observe increased levels of methyl sperm PP2A (Fig 4C). Significant methylation of PP2A by ABL127 was observed for proximal caput sperm that endogenously maintain high levels of demethylated PP2A. The distal caput and caudal sperm known to harbor higher levels of methylated form of PP2A showed only a small increase in methylation. The treatment of sperm with ABL127 however could not induce motility in proximal caput or distal caput sperm or affect motility characteristics of caudal sperm.

### Demethylation and reciprocal tyrosine phosphorylation of sperm PP2A by L-homocysteine and adenosine or okadaic acid

S-Adenosyl-L-methionine (SAM) is a universal methyl donor in methylation reactions. Following transfer of its methyl group SAM is converted to S-adenosylhomocysteine (SAH). Cellular SAH is broken down to homocysteine and adenosine by the enzyme SAH hydrolase. Homocysteine and adenosine are further metabolized or transported out of the cell. Elevated intracellular SAH is a feedback inhibitor of methyl transferases preventing methylation reactions. Incubation of cells including spermatozoa with adenosine and homocysteine results in increased intracellular SAH levels. L-homocysteine by itself also increases the intracellular SAH levels but to a lesser extent than when adenosine is also present [26].

**Fig 5:**
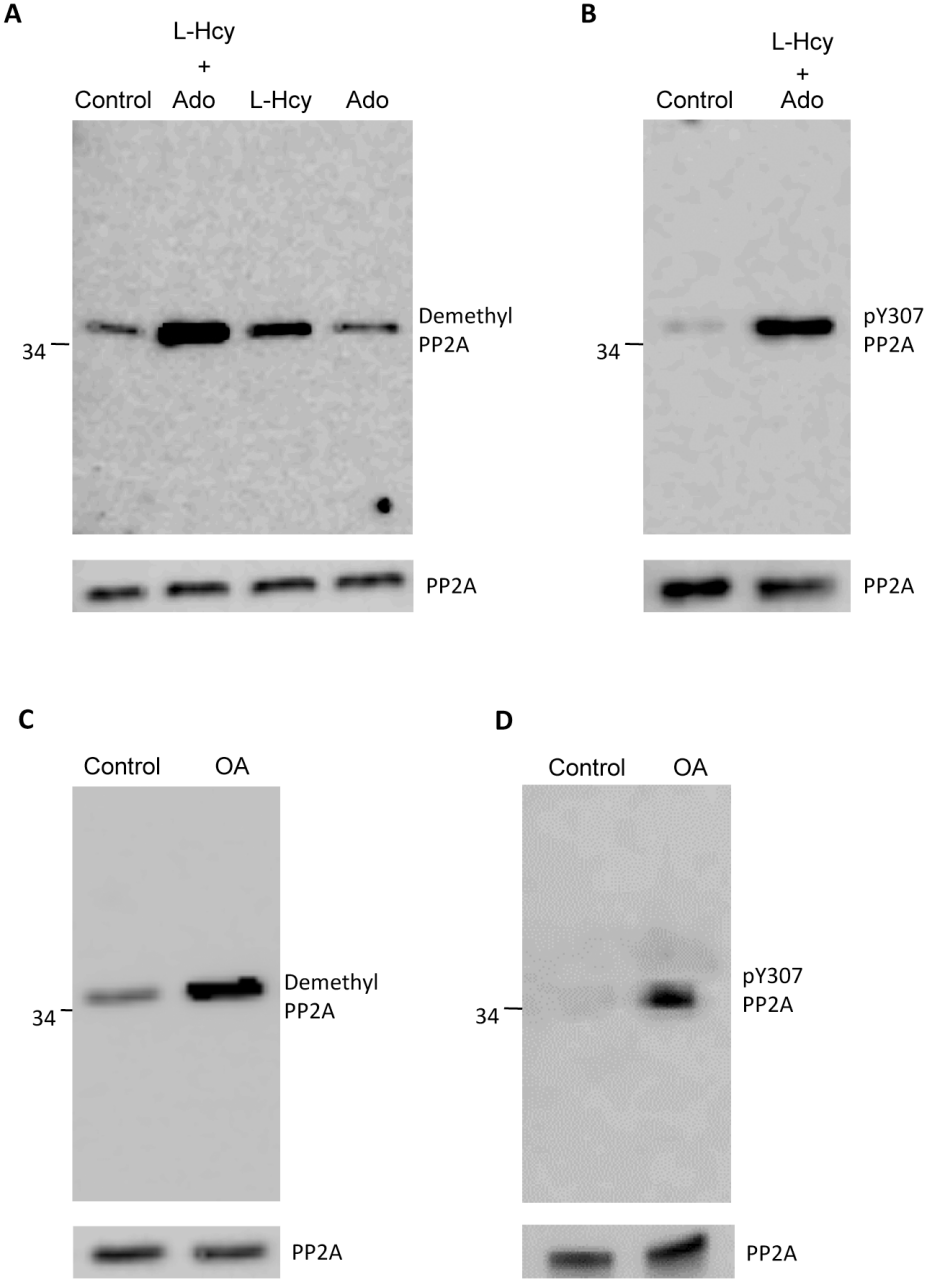
*In vivo* demethylation of sperm PP2A. **(A)** Caudal sperm were incubated with 1 mM each of L-Homocysteine and Adenosine (L-Hcy + Ado), 1 mM L- Homocysteine (L-Hcy) or 1 mM adenosine (Ado) for 10 min. Sperm extracts were prepared by sonication and analyzed by western blot (2X10^6^ sperm/lane) with anti-demethyl PP2A antibody and a duplicate blot probed with anti-PP2A antibody. Significant increase in levels of demethyl PP2A is observed on treatment with L-Homocysteine and adenosine (L-Hcy + Ado) compared to the untreated control sperm. (**B)** PP2A in extracts of L-Homocysteine and adenosine treated sperm was concentrated by microcystin pull down followed by western blot analysis of with anti-phosphotyrosine-PP2A (Y307) antibody shows increased levels of phosphorylated PP2A. (**C)** Caudal sperm incubated with 5 nM okadaic acid (OA), a concentration to specifically inhibit PP2A, also elevates demethyl PP2A and tyrosine phosphorylated PP2A as seen in **(D)** with 10^7^ sperm/lane.

Caudal spermatozoa (10^8^ /ml) were incubated at 37°C with 1 mM L-homocysteine with 1mM adenosine, 1 mM L-homocysteine alone, or 1 mM adenosine alone for 10 min in a suspension buffer containing glucose and BSA. Western blot analysis of extracts of sperm following this incubation is shown in Fig 5A. In a parallel experiment (Fig 5B), PP2A in extracts of untreated control sperm and sperm incubated in the presence of L-homocysteine and adenosine was concentrated by microcystin pull down followed by Western blot analysis with the anti-phosphotyrosine-PP2A (Y307) antibody. Increased PP2A concentration by microcystin pull down is required because of the lower affinity of the anti-phosphotyrosine-PP2A (Y307) antibody. Incubation with L-homocysteine and adenosine results in demethylation (Fig 5A) and a corresponding increase in tyrosine phosphorylation of PP2A (Fig 5B). Next we were curious to know as to how pharmacologic inhibition of PP2A may affect its methylation. We used okadaic acid (OA), which at nanomolar levels is known to inhibit PP2A but not PP1 [23]. Caudal sperm were incubated at 37°C with 5 nM okadaic acid in HEPES buffer supplemented with glucose and BSA. Western blot analysis of these sperm extracts show demethylation of PP2A presumably due to increased hydrolysis of the methyl group on Leu 309 of PP2A and increased PP2A tyrosine phosphorylation (Fig 5C and 5D).

### Catalytic activity of sperm PP2A decreases during passage through the epididymis

**Fig 6:**
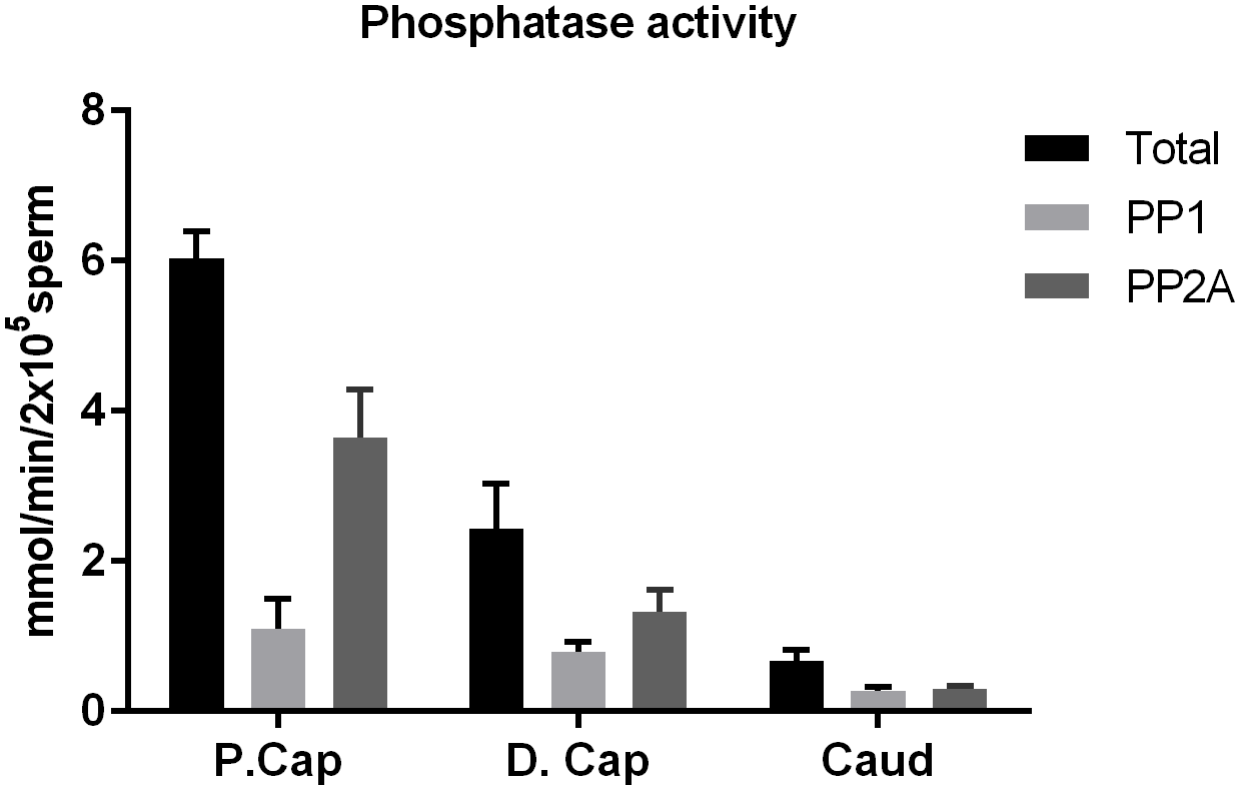
Protein phosphatase activity in sperm. Sperm extracts from proximal caput, distal caput and caudal regions of epididymis were prepared by sonication. The soluble fraction of the extracts that has been previously shown to contain the majority of sperm PP2A was analyzed for phosphatase activity with phosphorylase *a* as the substrate. The mean phosphatase activity (of n = 4 experiments each using different set of extract ±SEM) is expressed as mmol of PO4 released/minute/2x10^5^ sperm. The combined activity of PP1 and PP2A is shown as total phosphatase activity (black bars). PP2A activity was measured using 2nM OA (that is activity that was inhibited by 2nM OA) and PP1 activity by inhibition with I2 (activity that was inhibited by I2).

We measured catalytic activity of PP2A in sperm extracts using phosphorylase *a*, which is a substrate for both PP1 and PP2A [34]. The linear relationship of phosphatase activity with time and concentration of sperm extract are reported in our previous publications [23,24]. Similar results obtained from more recent experiments are shown in S1 File. Sperm extracts contain both PP1 and PP2A. PP2A is present in the soluble sperm extracts whereas PP1 is present both in the soluble and insoluble sperm extracts [35]. Focusing on the soluble fraction, as summarized in Fig 6, the total phosphatase activity expressed as a mean of n = 4 ± SEM in proximal caput, distal caput, and caudal sperm extracts was 6, 2.4 and 0.7 mmol of PO4 released/minute/2x10^5^ sperm, respectively. The heat-stable protein phosphatase Inhibitor-2 (I-2) inhibits PP1 (IC_50_ of 1 nM) but not PP2A [36]. Phosphatase activity that is inhibited by the inhibitor I-2 (PPP1R2) is due to PP1 and the portion of the activity inhibited by 2nM OA is attributed to PP2A. The mean PP2A activity (determined in four experiments) in proximal caput, distal caput, and caudal sperm extracts was 3.6, 1.3 and 0.3 mmol of PO^4^ released/minute/2x10^5^ sperm, respectively. As seen in Fig 6, PP2A activity in sperm from the proximal caput region is approximately three fold higher compared to sperm from the late caput region whereas it is twelve-fold higher compared to sperm from the caudal region of the epididymis.

### L-homocysteine and adenosine or OA treatment stimulates caudal sperm motility

Next we examined if demethylation of PP2A or PP2A inhibition could initiate sperm motility in distal caput sperm. Distal caput sperm were treated with 1 mM L-homocysteine and 1 mM adenosine to demethylate PP2A or 5 nM okadaic acid to inhibit PP2A. These treatments could not initiate motility in immotile caput epididymal sperm even when L-homocysteine and adenosine concentrations were increased to 10 mM and up to four hours of incubation. However, as previously reported, higher concentration of the okadaic acid (1 uM), a concentration that inhibits both PP1 and PP2A was able to initiate motility in immotile distal caput sperm [23].

**Fig 7:**
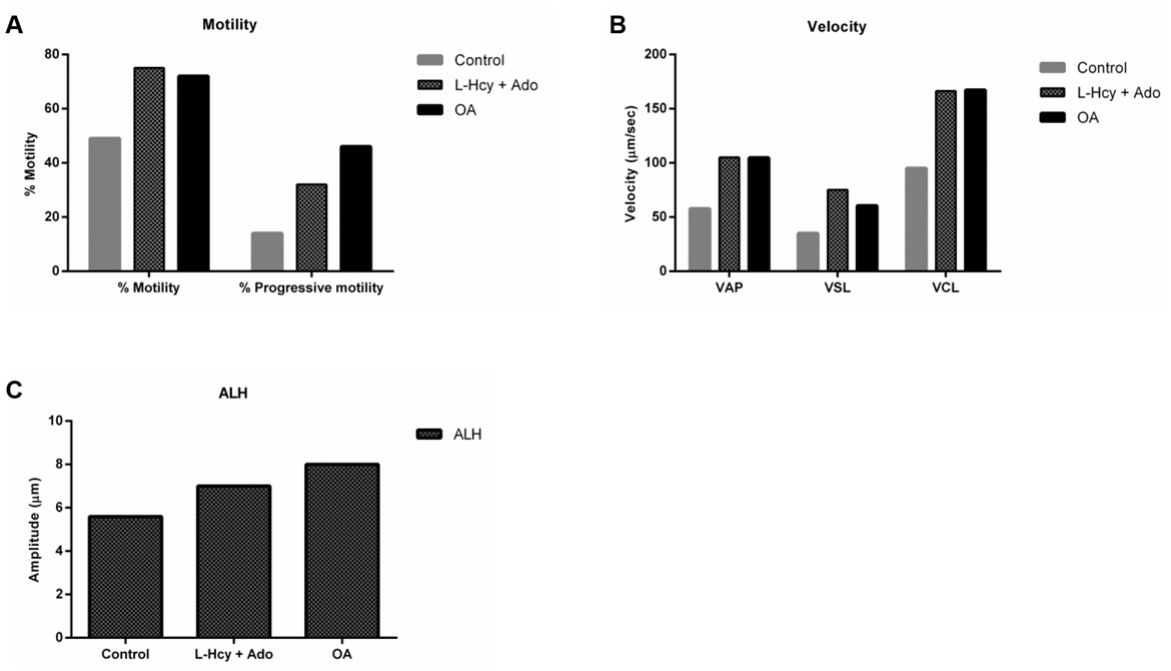
Computer assisted motility analysis of sperm. **(A)** Caudal sperm treated with 1 mM L-homocysteine and adenosine or 5 nM okadaic acid show increased percentage motility and progressive motility after 10 min of incubation. **(B, C)** Sperm velocity parameters such as path velocity (VAP), Straight line velocity (VSL), Track speed (VCL) and lateral amplitude (ALH) are also increased compared to the control. The data shown is a representation of nine similar experiments.

**Fig 8:**
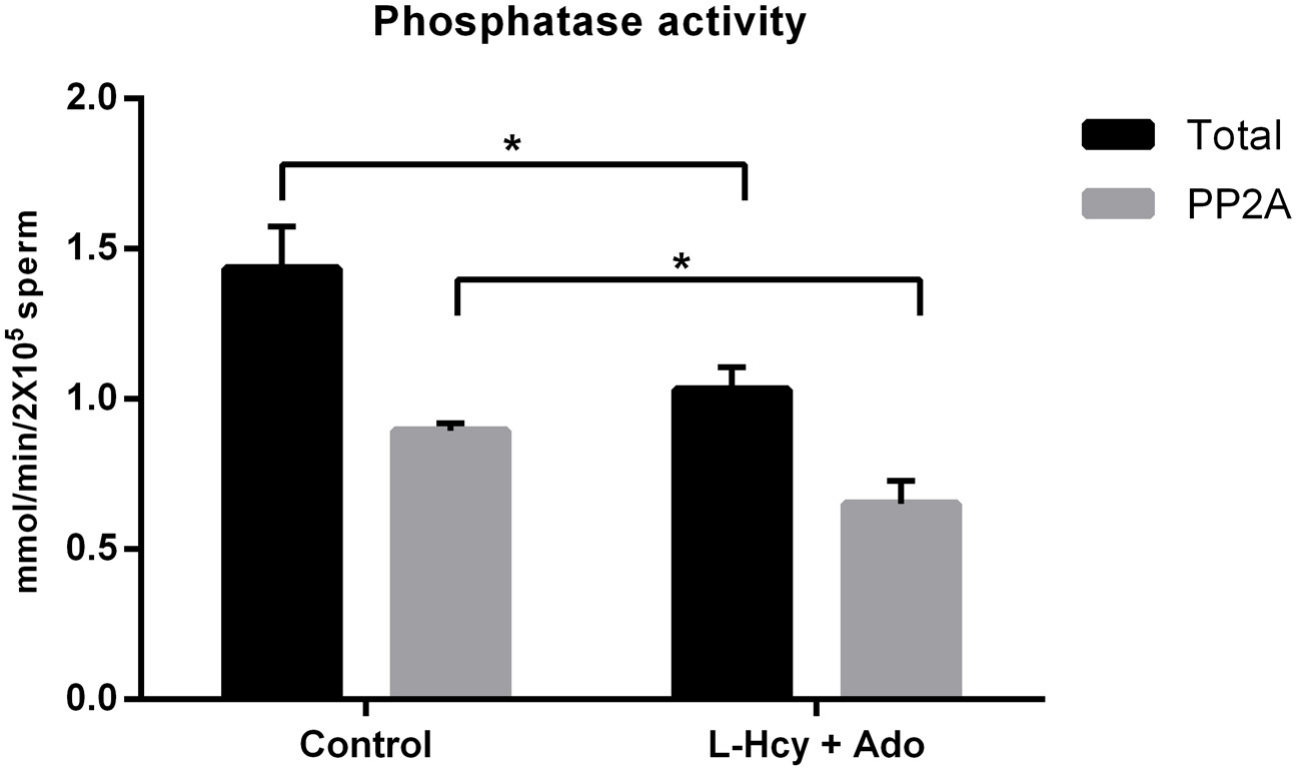
Catalytic activity of PP2A following its demethylation by L-homocysteine and adenosine treatment. Caudal sperm incubated with 1 mM L-homocysteine and adenosine were sonicated and the soluble protein fraction was collected. Protein phosphatase activity in this fraction was measured with phosphorylase *a* as the substrate. PP2A activity was measured as the activity that can be inhibited by 2nM OA. Demethylation of sperm PP2A by L-homocysteine and adenosine results in decreased total phosphatase and PP2A catalytic activity. The mean phosphatase activities from five sets of experiments are represented as mmol of PO4 released/minute/2x10^5^ sperm ± SEM. ‘*’ denotes significant difference with P< 0.05. The demethylation in each experiment was confirmed by western blot analysis of the sperm extract.

While treatment with L-homocysteine and adenosine or okadaic acid did not stimulate motility in caput sperm, these compounds could affect the motility of caudal sperm. Caudal epididymal sperm were incubated with 5 nM okadaic acid or 1 mM L-homocysteine and 1mM adenosine in HEPES buffer supplemented with glucose and BSA at 37^°^. Motility was quantified with a Computer Assisted Sperm motility Analyzer (CASA) system. For both the L-homocysteine and adenosine- treated and okadaic acid-treated sperm, all motility parameters were increased compared to control (S2 File). The percent motility, path velocity (VAP), track speed (VCL), progressive velocity (VSL) and lateral amplitude (ALH) were increased. Results obtained from one of nine similar experiments are shown in Fig 7. The motility stimulation by L-homocysteine and adenosine was surprising suggesting that increased levels of S-adenosylhomocysteine inside the sperm cell may have decreased rather than increased phosphatase activity. Indeed, phosphatase activity measurement of L-homocysteine and adenosine-treated sperm had lowered PP2A activity compared to control sperm (Fig 8).

### Relationship between PP2A and GSK3 phosphorylation

**Fig 9:**
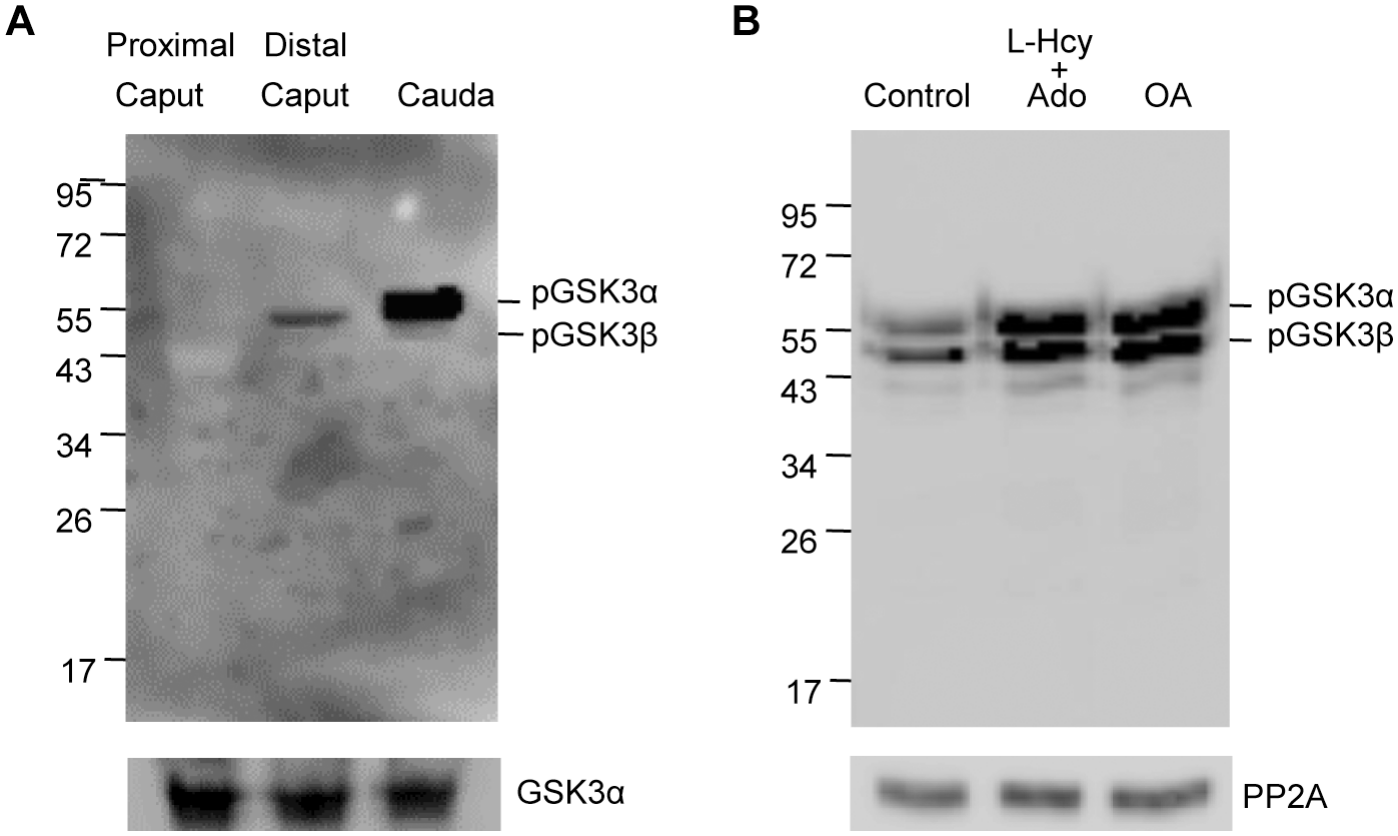
Serine phosphorylation of GSK3α/β. **(A)** Western blot analysis with anti-phospho GSK3α/β (pSer 21/9) antibody of freshly prepared sperm extract (4X10^6^ sperm/lane) from sperm of proximal caput, distal caput and caudal regions of epididymis. **(B)** Sperm incubated at 37°C in 1 mM L-homocysteine and adenosine or 5 nM okadaic acid in HEPES buffer supplemented with glucose and BSA show increased immunoreactivity at 55Kd and 47Kd corresponding to phosphorylated GSK3α and GSK3β respectively (2X10^6^ sperm/lane). The same blot was re-probed with Anti-PP2A antibody showing equal protein loading.

**Fig 10:**
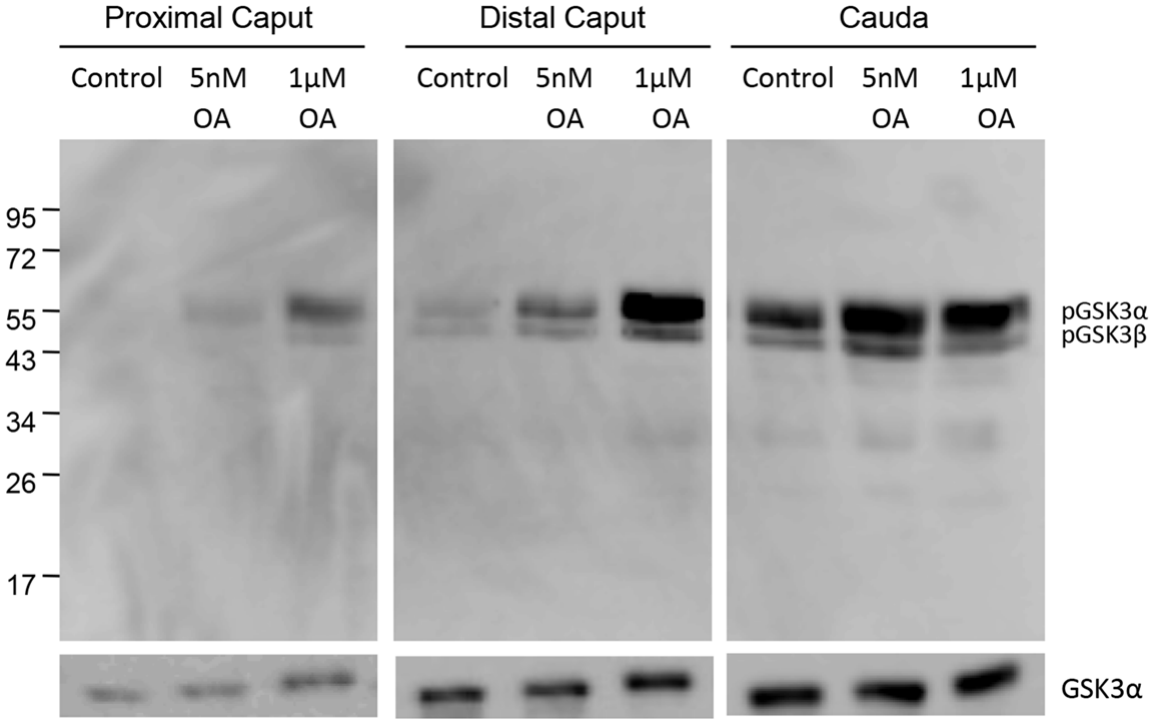
Effect of PP2A or PP1 inhibition of serine phosphorylation of GSK3. Sperm isolated from proximal caput, distal caput and cauda were treated with either 5nM OA to specifically inhibit PP2A or 1uM OA to inhibit both PP1 and PP2A. The extracts were analyzed by western blot analysis with anti-phospho GSK3α/β (pSer 21/9) antibody. 10^6^ proximal caput sperm/lane and 4X10^6^ distal caput or caudal sperm/lane was loaded.

Increased glycogen synthase kinase 3 (GSK3) phosphorylation is associated with sperm maturation and motility [23]. It appears that phospho GSK3 is a substrate of PP2A [37]. We investigated whether dephosphorylation of GSK3 could also be mediated by PP2A in sperm. As shown in Fig 9A, GSK3 is virtually unphosphorylated in proximal caput sperm. GSK3 phosphorylation is observed in sperm from distal caput epididymis reaching high levels in caudal epididymal sperm. Further increases in GSK3 phosphorylation in caudal sperm occurs following incubation with L-homocysteine and adenosine or okadaic acid (Fig 9B), treatments expected to inhibit PP2A. To distinguish between the roles of PP2A and PP1 on GSK3 phosphorylation, sperm from the three regions of the epididymis were incubated with 5nM OA or 1uM OA. PP2A but not PP1 is expected to be inhibited with 5 nM OA whereas both PP1 and PP2A are expected to be inhibited with 1 uM OA. Western blot analysis (Fig 10) show that in proximal and distal caput epididymal sperm GSK3 is serine phosphorylated to a greater extent in the presence of 1uM compared to 1 nM OA. In caudal sperm however, increased GSK3 phosphorylation occurs with either 1nM or 1uM OA (Fig 10). That is, inhibition of PP2A appears to be sufficient to increase GSK3 phosphorylation in caudal but not in proximal and distal caput epididymal sperm.

## Discussion

We previously identified that PP2A is one of the two serine/threonine phosphatases in sperm. The other is PP1γ2, which is present both in the soluble and insoluble fractions of sperm sonicates [38]. Here we show that sperm PP2A is almost entirely cytosolic, with negligible amounts in the insoluble fraction of sperm sonicates. Western blot analysis, using a demethyl-sensitive PP2A antibody, indicates that PP2A is in its demethylated form in sperm from the proximal caput region of epididymis. As sperm mature and progress to the distal caput region, PP2A undergoes methylation on its carboxy terminus with a corresponding reduction in tyrosine phosphorylation. These changes in the methylation state of PP2A were deduced by the use of the demethyl-sensitive and methyl-insensitive PP2A antibodies. PP2A in sperm from distal caput and caudal regions underwent an apparent increase in methylation resulting in decreased immunoreactivity to the demethyl-sensitive antibody. The methylation status of PP2A was further confirmed by demonstrating that microcystin-bound PP2A can be demethylated by alkali treatment. Proximal caput sperm PP2A was unaffected by alkali treatment suggesting it was in its demethyl form. It may be noted that the proportion of the PP2A protein that is methylated in distal caput and caudal epididymal sperm is not known.

Reversible methylation of PP2A requires the presence of leucine carboxyl methyl transferase 1 (LCMT1) and PP2A specific methyl esterase 1 (PME1). The fact that PP2A methylation changes *in vivo* and *in vitro* (Figs 1 and 5) also implies the presence of LCMT1 and PME1 in sperm. Western blot analysis of testis and sperm extracts with anti-LCMT1and anti-PME1 antibodies showed immunoreactive bands corresponding to the molecular weight of LCMT1 and PME1. Higher levels of PME1 that was observed could be the possible explanation for predominantly demethylated pool of PP2A in proximal caput sperm. The universal methyl donor in methylation reactions is S-adenosyl methionine (SAM). Following donation of the methyl group SAM is converted to S-adenosylhomocysteine (SAH). SAH in turn can be hydrolyzed to homocysteine and adenosine by the enzyme SAH-hydrolase. Intracellular SAH is a feedback inhibitor of methylation. Intriguingly, SAH-hydrolase contains an allosteric binding site for cAMP, which activates the enzyme [39,40]. This action of cAMP on SAH-hydrolase activity would be independent of PKA activation. The ability of PP2A to associate with a variety of regulatory subunits and/or the essential requirement for reversible methylation of the catalytic subunit suggests—multiple mechanisms for regulating PP2A activity in cells.

Catalytic activity is high when PP2A is in its demethylated form in immature proximal caput epididymal sperm. Increased PP2A methylation lowers PP2A catalytic activity as sperm traverse the epididymis. Given this situation *in vivo*, it is puzzling that the activity of PP2A is lowered, rather than increased, in caudal epididymal sperm following incubation of sperm with adenosine and homocysteine *in vitro* which results in demethylation of PP2A. It is possible that SAH is both an inhibitor of methyl transferase and of PP2A activity. It is interesting in this regard that pharmacological inhibition with okadaic acid also causes demethylation accompanied by increased tyrosine phosphorylation of PP2A (Fig 5C and 5D). Indirect evidence correlating increased SAH levels with increased tau-phosphorylation in the brain may support the notion that SAH may also inhibit PP2A catalytic activity [41]. Alternatively it is possible that demethylated PP2A is less active in caudal epididymal sperm by virtue of the differences in the composition of the PP2A holoenzyme in early caput compared to caudal sperm. For example, PP2A methylation is not required for formation of hetero-trimer with regulatory subunits belonging to the B‴ family but methylation is necessary for trimer formation with B' family of subunits[42]. The activity of these different hetero timer permutations or differing levels of PP2A-C incorporated into these trimer complexes depending on the regulatory subunits could have variable effect on the phosphorylase *a* substrate used to measure PP2A activity possibly explaining the observed discrepancies. Virtually nothing is known about the components of the PP2A holoenzyme in sperm. Formation of the PP2A holoenzyme is one of the consequences of PP2A methylation. Further insights into the catalytic activity of PP2A in relation to its methylation must await information about the regulatory proteins associated with PP2A in developing sperm.

We tried to increase methylation of PP2A in sperm by selectively inhibiting PME1 with ABL-127 to determine how increased PP2A methylation will affect sperm motility. As expected we observe a significant increase in levels of methyl PP2A in proximal caput sperm that contain predominantly the demethylated form of PP2A and high levels of PME1 as detected on Western blot. We saw only a minor effect on PP2A in sperm from distal caput or caudal epididymis. However, the sperm motility parameters were unaffected following this treatment of sperm from any of the three regions of epididymis. Our efforts to increase PP2A methylation in caput epididymal sperm by increasing intracellular SAM levels following incubation with methionine and adenosine were unsuccessful. Why and how methylation occurs *in vivo* during sperm maturation and whether sperm contain sufficient levels of intracellular SAM is not known.

A series of studies in the 1980s with human, mouse and hamster sperm suggested that protein carboxy-methylation was involved in sperm motility [26,43–47]. At that time, the identities of the macromolecules modified by methylation in sperm were not known. The assays for methylation in these early reports used BSA as a methyl acceptor. We suggest that the results of these early studies can be re-interpreted in light of our discovery that sperm PP2A is regulated by methylation. It is possible that the motility effects observed in these earlier studies attributed to changes in sperm protein carboxy-methylation might well be due to PP2A regulation.

The down-stream targets of PP2A are yet to be determined. We suggest that one of the substrates of sperm PP2A is the signaling enzyme glycogen synthase kinase 3, GSK-3. The role of PP2A in dephosphorylating GSK3 in somatic cells is documented [37]. We have previously shown that during epididymal initiation of sperm motility, GSK-3 is phosphorylated on a serine residue leading to a reduction in its catalytic activity [48]. It is possible that the high catalytic activity of PP2A in immotile distal caput sperm may be one of the factors responsible for the low levels of GSK-3 phosphorylation. Conversely the lower catalytic activity of PP2A in caudal epididymal sperm may be responsible for the higher steady state levels of GSK3 phosphorylation. A potential substrate of GSK-3 in epididymal sperm is the PP1 regulatory subunit, PPP1R2 (inhibitor I-2) [23,49]. We have suggested that reversible phosphorylation of the inhibitor I-2 may be involved in regulating sperm PP1γ2. Interestingly, the PP1γ2—I-2 complex is almost exclusively localized in the insoluble fraction of sperm extracts suggesting that the complex may be bound to structures in the axoneme. Changes in PP2A activity may influence PP1γ2 activity in the flagellum through its regulation of GSK3. We have recently shown that GSK3α isoform has an essential role in male reproduction and its ablation from testis results in abnormal sperm motility resulting in male infertility [35]. Catalytic activity of PP1γ2 is higher in sperm lacking GSK3α compared to sperm from wild type mice [35].

GSK3 is either a direct or an indirect substrate of PP2A because caudal epididymal sperm treated with either 1 mM L-homocysteine and adenosine or 5 nM OA resulted in increased phosphorylation of GSK3α/β. Demethylation of PP2A induced by 1 mM L-homocysteine and adenosine or 5 nM OA could not initiate motility in caput sperm. That is, lowering PP2A activity alone is not sufficient for initiation of sperm motility. We have previously shown that motility initiation requires conditions that inhibit PP1 [23]. However in motile sperm, inhibition of PP2A, either by L-homocysteine and adenosine or OA leads to significant stimulation of sperm motility. It appears that motility patterns exhibited when PP2A is inhibited resemble sperm hyperactivation. Others have made similar observations with hamster sperm using differential inhibition of PP1 and PP2A [50]. Whether PP2A undergoes changes in its methylation and catalytic activity prior to fertilization merits further investigation.

In summary, our data show that PP2A is present in bovine spermatozoa and document for the first time that changes in its catalytic activity associated with its demethylation, occur during passage of sperm through the epididymis. Inhibition of PP2A while not sufficient for motility initiation results in motility stimulation in sperm that are already motile. One of the targets of PP2A is GSK3 since its phosphorylation is increased by PP2A inhibition. The relationship between PP2A, GSK3 phosphorylation, and PP1γ2 activity in regulating sperm function during initiation of sperm motility and during fertilization of eggs is being investigated in our laboratory.

## Supporting Information

S1 File Standardization of phosphatase activity in sperm extracts. **(Fig A)** Sperm extracts from caudal regions of epididymi were prepared by sonication. The soluble fraction of the extracts was serially diluted and analyzed for phosphatase activity with phosphorylase *a* as the substrate. A 4ul sperm extract aliquot initially containing 4X10^6^ sperm were serially diluted to 0.25X10^6^ sperm. The combined activity of PP1 and PP2A denoted in mmol/min decreases in a linear fashion with decreasing sperm numbers in the extract. **(Fig B)** A caudal sperm concentration of 2x10^6^/ml was further used to measure phosphatase activity at various time points with intervals of approximately 3mins. The total phosphatase activity shown in counts per minute (CPM) increases linearly with time. The 10 minute time point was used in all our experiments.
(TIF)

S2 File Sperm motility videos. CASA recordings of Bovine sperm in HEPES buffer supplemented with glucose and BSA **(Fig A)** without any treatment (Control), **(Fig B)** treated with 1mM L-homocysteine and adenosine (L-Hcy + Ado) and **(Fig C)** 5 nM okadaic acid (OA) after 10 minutes of incubation at 37°C. Sperm treated with 1mM L-homocysteine and adenosine or 5nM okadaic acid show increased percentage motility, velocity and lateral head amplitude.
(WMV)
