# Supplementary figures and images for: Changes in Carboxy Methylation and Tyrosine Phosphorylation of Protein Phosphatase PP2A Are Associated with Epididymal Sperm Maturation and Motility
Source: PLoS One. 2015 Nov 16;10(11):e0141961. doi: 10.1371/journal.pone.0141961 (PMC4646675; doi:10.1371/journal.pone.0141961)

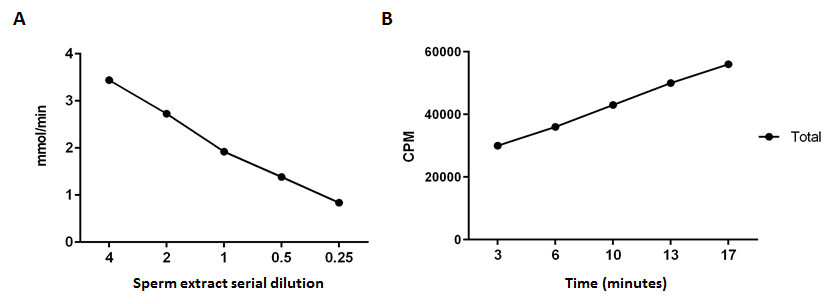

Supplement: S1 File — (Fig A) Sperm extracts from caudal regions of epididymi were prepared by sonication. The soluble fraction of the extracts was serially diluted and analyzed for phosphatase activity with phosphorylase a as the substrate. A 4ul sperm extract aliquot initially containing 4X106 sperm were serially diluted to 0.25X106 sperm. The combined activity of PP1 and PP2A denoted in mmol/min decreases in a linear fashion with decreasing sperm numbers in the extract. (Fig B) A caudal sperm concentration of 2x10^6^/ml was further used to measure phosphatase activity at various time points with intervals of approximately 3mins. The total phosphatase activity shown in counts per minute (CPM) increases linearly with time. The 10 minute time point was used in all our experiments. (TIF) [file pone.0141961.s001.tif]
